# Advances in single-cell transcriptomic sequencing in hepatic echinococcosis

**DOI:** 10.3389/fimmu.2026.1829801

**Published:** 2026-07-13

**Authors:** Yi-bin Xie, Chun-xiu He, Qin-dong Jing, Rui-long Ma, Yu-qiang Tang, Lin-xun Liu, Ji-de A, Qing-long Tan

**Affiliations:** 1 School of Clinical Medicine, Qinghai University, Xining, China; 2 Department of General Surgery, Qinghai Provincial People’s Hospital, Xining, China; 3 Department of General Surgery, Pingliang Second People’s Hospital, Pingliang, China; 4 Department of Hepatic Hydatidosis, Qinghai Provincial People’s Hospital, Xining, China

**Keywords:** growth and interaction, hepatic echinococcosis, heterogeneity, molecular diagnostics, single-cell transcriptomic sequencing, transfer

## Abstract

*Echinococcosis* is a chronic parasitic disease and a serious zoonotic infection caused by the adult or larval stages of Echinococcus tapeworms. The transmission of *Echinococcosis* depends on carnivores as definitive hosts and various animals as intermediate hosts. According to current epidemiological reports, the liver is the most commonly affected organ. Clinically, Hepatic *Echinococcosis* is classified into hepatic alveolar *Echinococcosis* and hepatic cystic *Echinococcosis*, both of which pose a serious threat to the quality of life of residents in endemic areas. Currently, surgical treatment combined with benzimidazole medication is the main therapy; however, there are few chemotherapeutic drugs and targeted treatments available, and clinical treatment still has limitations. Therefore, there is an urgent need for new exploration in the molecular immunology, cellular directions. In recent years, with the development of high-throughput sequencing technology, integrated analyses of spatial transcriptomics and single-cell transcriptomics have become more accessible in clinical research, providing new approaches for the study of hepatic *Echinococcosis*. This review focuses on the application of single-cell RNA sequencing in hepatic*Echinococcosis* in recent years, as well as research on molecular immunopathogenesis, moleculardiagnostics, metastasis, cellular heterogeneity, growth and development, and the interactions between human hosts and echinococcosis. We pay particularly attentionto the heterogeneity of infiltrating immune cells around lesions, classification of cell subpopulations, immune suppression, and the immune microenvironment, which can help clinicians better understand the molecular immune mechanisms and biological characteristics of hepatic *Echinococcosis*, providing references for exploring new drug therapeutic targets.

## Introduction

**Figure 1:**
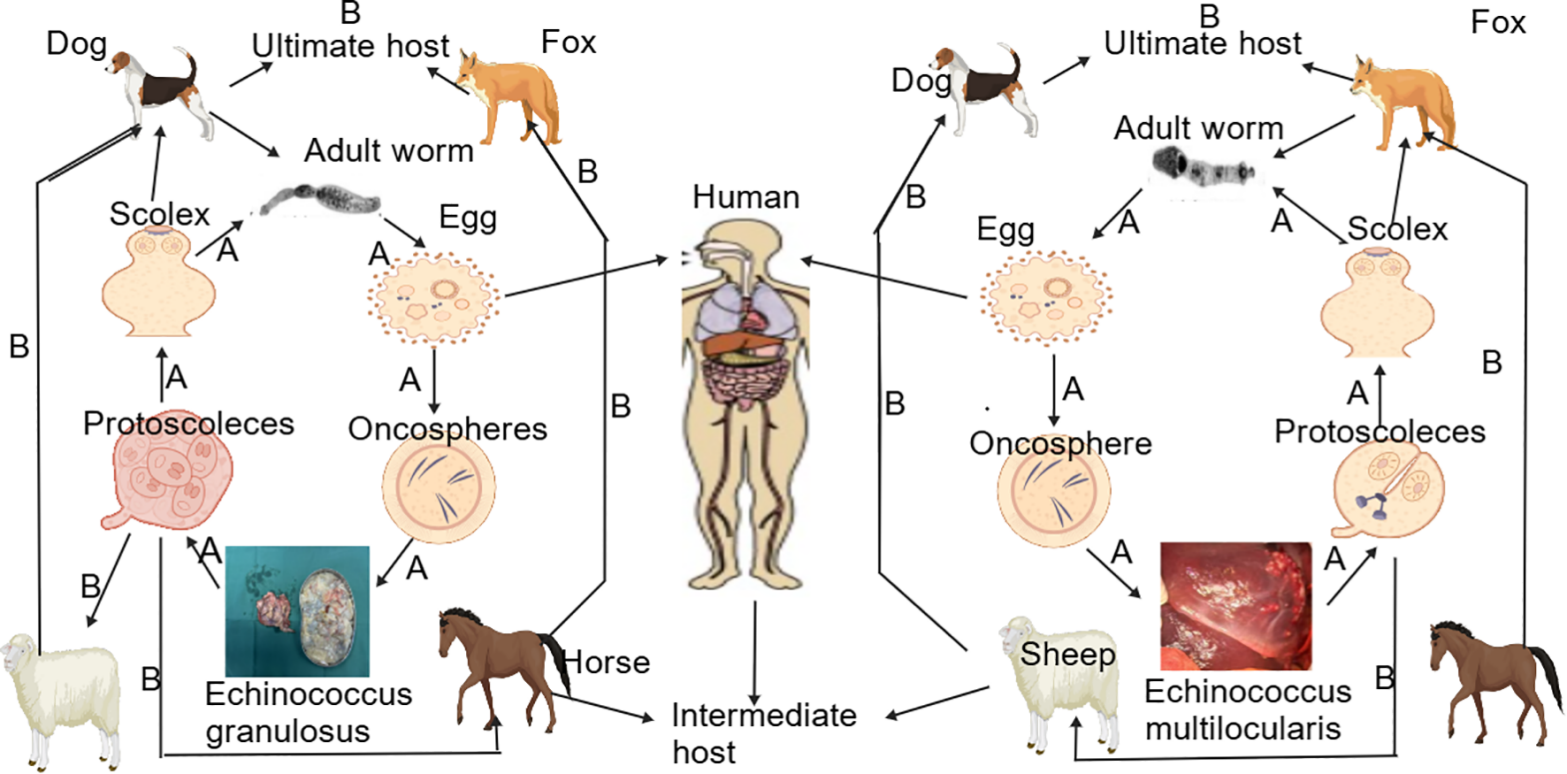
Life cycles of Echinococcus multilocular and Echinococcus granulosus. **(A)** Host transmission routes of Echinococcus granulosus and Echinococcus multilocularis. **(B).**.

*Echinococcosis*, a chronic parasitic disease, is a severe zoonotic disease caused by the adult or larval stages of the *Echinococcus* tapeworm (1).The propagation of echinococcosis depends on carnivores as the definitive host and various animals as intermediate hosts (2). Infection occurs when *Echinococcus* eggs are ingested either directly or indirectly from materials contaminated with feces of infected carnivores (3). Clinically, hepatic echinococcosis is classified into hepatic alveolar echinococcosis and hepatic cystic echinococcosis, both of which pose a significant threat to the quality of life of residents in endemic areas (4). (**Figure 1**) In recent years,high-throughput sequencing technology has advanced rapidly, and single-cell sequencing has shownsignificant advantages over traditional sequencing methods in addressing problems such as biologicalheterogeneity and scarcity of biological materials. This has greatly revolutionized our understanding ofmany biological phenomena, such as gene transcription, embryonic development, and carcinogenesis (5). Single-cell RNA sequencing (ScRNA-seq, SCS) (**Table 1**) is a powerful tool for defining thecell type and state of individual cells associated with health and disease (6). Recently,SCS has beenused to analyze infiltrating lymphocytes in cancer (7, 8) and cystic echinococcosis CE (**Table 1**) (9). SCS provides insights into the dynamic nature of these invasive cells in highly complex cancer orparasitic microenvironments. Furthermore,SCS is an advanced method for defining the T cell receptor (TCR) sequence of a single-cell (10). Its application in the field of hepatic echinococcosis has Shown outstanding advantages in the diagnosis and pathogenesis of echinococcosis, as well as in theinteraction between the host and the immune microenvironment of echinococcal lesions. Echinococcosis consists primarily of CE and AE (**Table 1**) two serious zoonotic forms of tapeworms caused by *Echinococcus granulosus* and *Echinococcus multilocularis*, respectively (11).CE is a common worldwide echinococcosis that has been eradicated in some island nations (12, 13) It is a long-lasting disease that primarily affects the liver, followed by the lungs and the spleen (11, 14). Cysts grow expansively in organs, directly squeezing and replacing normal tissues, and long-term compression leads to ischemia, atrophy, and necrosis of local tissues.

**Table 1:** List of acronyms.

Abbreviation	Full-form
SCS	single-cell sequencing
BMZ	Benzimidazoles
AE	Alveolar echinococcosis
PT	Percutaneous therapy
CE	Cystic echinococcosis
PL	Liver periphery of the lesion
AN	Intraoperative fresh adjacent to normal liver
pDC	Plasmacytoid dendritic cells
SPP1	Secreted phosphorylated glycoprotein 1
PB	Preoperative peripheral venous blood
IL-C2	Type 2 congenital lymphocytes
cfDNA	Cell-free DNA
CRISPR	Clustered Regularly Interspaced Short Palindromic Repeats
DALYs	disability-adjusted life years
R&D	Research and Development
CLT	CD8+ T cells in the near liver tissue
OCR	oxygen consumption rate
ROS	reactive oxygen species
lncRNAs	long non-coding RNAs

AE is a potentially fatal parasitic disease caused by larval infection with the Northern Hemisphere tapeworm Echinococcus multilocularis. It is mainly transmitted by the ingestion of parasitic eggs, which originate in the definitive host, the canine, and are excreted in the feces. Asthe disease progresses, the invaded organs deteriorate, leading to organ dysfunction, failure, and ultimately, death (15). Also known as parasitic cancer, AE is the most lethal parasitic zoonosis, with a mortality rate of more than 90% over a 10-year period if left untreated (15). Also known as parasitic cancer, AE is the most lethal parasitic zoonosis, with a mortality rate of more than 90% over a 10-year period if left untreated (16). Furthermore, AE is becoming a major global public health problem and economic burden (17–19). In the natural ecological cycle, the life cycle of Echinococcus multilocularis exists between canines (final hosts) and small mammals, such as rodents and humans (intermediate hosts) (20). Eggs excreted by canines can contaminate the surrounding environment, and larvae develop in the body of an intermediate host when they are accidentally ingested during foraging or other daily activities. When canines prey on these infected intermediate hosts, the parasites further develop into adults in the canine body, thereby completing the life-cycle and creating potential conditions for the spread of AE (21).

CE is a zoonotic disease caused by the larvae of the tapeworm Echinococcus granulosus, which is a complex of 10 genetic species, namely, G1–G9 (Echinococcus granulosus sensu lato), and lion strains. Among them, the genetic species G1–G3 (Echinococcus granulosus sensu stricto) are closely related to each other and are collectively named Echinococcus granulosus (22, 23). The complexity of their structure has also posed many challenges and research on CE prevention and control. According to data published by the WHO, the global burden of disease in CE averages 285,500 disability-adjusted life years (DALYs) (**Table 1**), which could exceed 1 million DALYs if underreporting is factored; furthermore, the disease burden for AEs is 666,434 DALYs. These data reveal that CE and AE have placed a huge burden on the global health landscape and economic structure (14, 24). It is mainly manifested as lesions that occupy space, and the main risk is that rupture leads to allergic reactions, and its harm and damage to the body mainly stem from the mechanical mass effect of hydatid cysts, causing organ function to be impaired: such as liver compression leading to liver atrophy and abnormal liver function; Difficulty breathing, coughing due to pressure on the lungs; Compression of the brain leads to increased intracranial pressure and neurological dysfunction (headache, epilepsy, paralysis, etc.). obstructive jaundice caused by biliary compression or cyst rupture into the bile duct; portal hypertension due to portal vein compression (ascites, splenomegaly, esophageal and gastric varices); bronchial compression leading to atelectasis, obstructive pneumonia; Pressure on the ureters leads to hydronephrosis. Compression of the nerve or stretching of the capsule causes persistent or intermittent pain.

At present, echinococcosis is mainly treated with surgery, supplemented by benzimidazole drug therapy (BMZ) (**Table 1**). CE is supplemented by percutaneous therapy (PT) (**Table 1**) and benzimidazole drug therapy (BMZ), among which albendazole is currently the most widely used drug in the world, and the combination with surgery has the best efficacy. AEsare mainly treated with surgery, supplemented by medical treatment with benzimidazole (BMZ) (25, 26). In recent years, vaccine R&D (**Table 1**) has made great strides, bringing good prospects for disease prevention (27–29). The vaccine for echinococcosis in animals has been successfully launched, while the vaccine for echinococcosis in humans is still in the research and development stage.

## Result

### 1 Single-cell RNA sequencing

**Figure 2:**
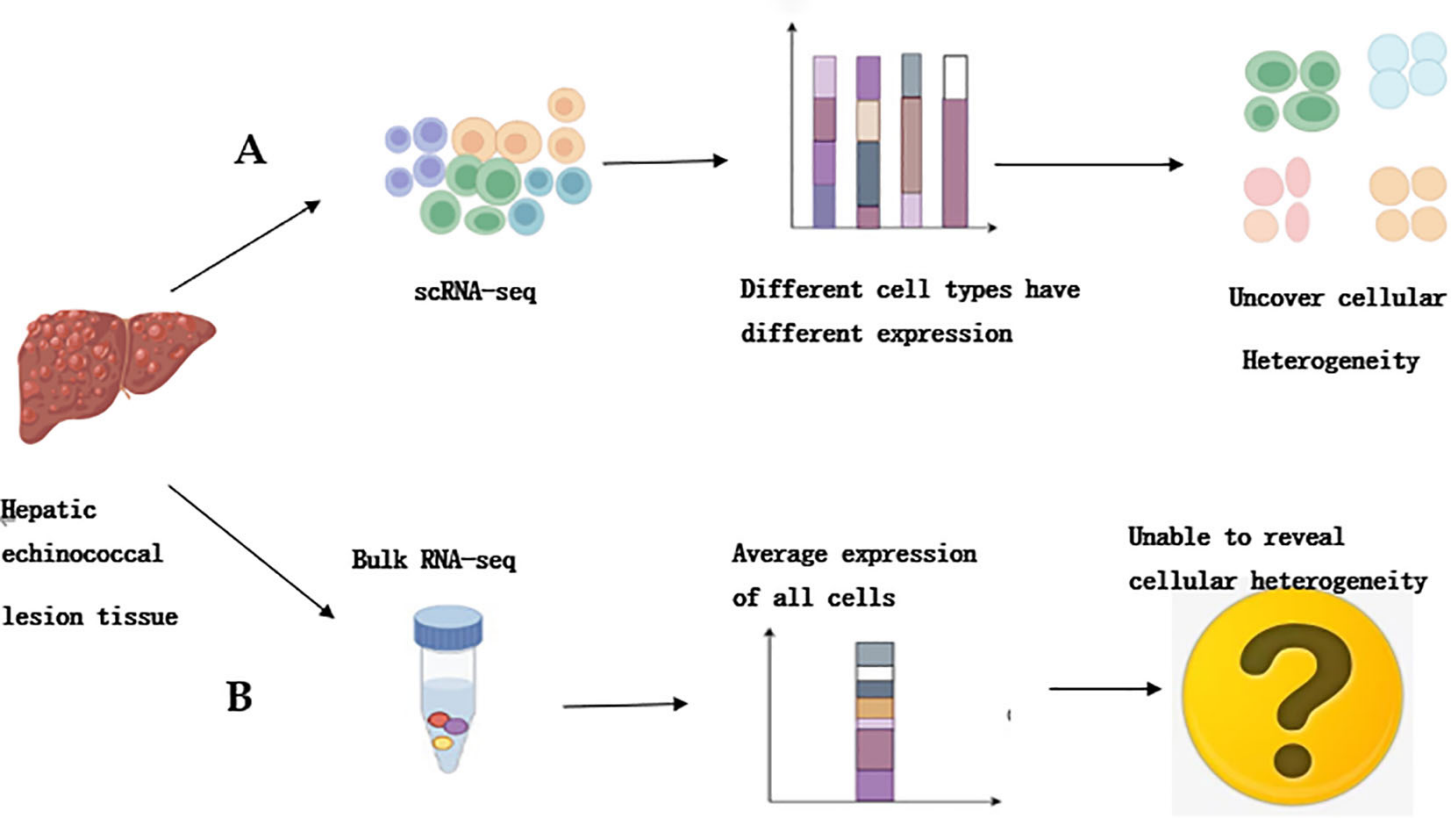
Comparison plot of scRNA-seq and bulk RNA-seq approaches. **(A)** scRNA-seq. scRNA-seq can reveal cellular heterogeneity. **(B)** Bulk RNA-seq cannot reveal cellular heterogeneity.

Bulk RNA sequencing (bulk RNA-seq) is a classic sequencing technology whose core process involves extracting RNA from a sample of cells or tissues and mixing all RNA for sequencing. However, compared to SCS(scRNA-seq), there are significant differences between the two methods in terms of the analytical scale and detection sensitivity. Bulk RNA-seq provides an average expression profile of the cell population, whereas scRNA-seq can reveal cellular heterogeneity at single-cell resolution (30) (**Figure 2**).

Compared to traditional bulk RNA-seq technology,SCS has the unique advantage of being able to effectively identify and characterize rare cell populations or heterogeneous cell subsets that can be easily masked in batch sequencing through a detailed analysis of individual cells (31). This high-resolution analytical method is a powerful tool for uncovering the complexity and diversity of cell populations. The standard workflow for SCS involves the isolation of individual cells, followed by RNA extraction from the cells, conversion of RNA to cDNA through reverse transcription, pre-amplification to increase the amount of cDNA, and analysis and detection of gene expression using highly sensitive assays (32). For large-scale sample sequencing, the high cost of scRNA-seq has become a challenging problem for many researchers and has hindered research advancement in this field. To overcome this problem, researchers have successfully built single-cell gene expression profiles by integrating scRNA-seq data and performing deconvolution analysis of bulk RNA-seq data using a reference spectrum (33). However, the deconvolution approach is not without flaws, and its effectiveness relies heavily on scRNA-seq reference data. If the reference data do not match the target sample or if there are errors in the cell annotation in the reference data, these factors can significantly reduce the accuracy and reliability of deconvolution (34). The latest batches of online tools developed based on deconvolution algorithms include MuSiC2 (35), TIMER2.0 (36), BayesPrism (37), Coex and Bisque (38). Moreover, at present, single-cell genomics and spatial transcriptomics each have their own advantages, Combining the two, in new multi-omics applications, you can take advantage of both and apply them in future genomics research (**Table 2**).

**Table 2:** Advantages and disadvantages of commonly used single-cell transcriptome sequencing and spatial transcriptome sequencing.

Platform	Separation method	Amplification method	Amplification range	Advantages	Disadvantages	References
Smart	FACS	PCR	Full-Length	High single-cell coverage and genetic analysis ability.	Insensitive to low-abundance transcripts, cost high.	(101)
Smart-seq2	FACS	PCR	Full-Length	Improved transcriptome coverage and accuracy.	Limited information capture.	(102)
Smart-seq3	FACS	PCR	Full-Length	Higher sensitivity and coverage.	The cost is higher and the technology is more complex.	(103)
CEL-seq	FACS	IVT	3′ end	Good reproducibility and high sensitivity.	Low throughput and amplification efficiency.	(104)
Tang-2009	FACS	PCR	3′ end	Good reproducibility.	High cost and low efficiency.	(105)
CEL-seq2	FACS	PCR	3′ end	High sensitivity and low cost.	The library is biased towards the 3′ end of the gene, with high abundance transcript priority amplification.	(106)
10X-Genomics	Droplet	PCR	3′ end	High cell capture efficiency and short cycle time, good reproducibility.	Only the 3′ end can be sequenced.	(107)
Drop-seq	Droplet	PCR	3′ end	Fast, high-throughput, and cost-effective.	High requirements for single-cell suspensions.	(108)
inDrop-seq	Droplet	IVT	3′ end	Low cost and linear scale-up.	The operation time is long and the sample requirements are high.	(109)
MARS-seq	FACS	In vitro transcription	3′ end	Good reproducibility and effective control of deviations.	The experimental operation and data processing requirements are high.	(110)
MARS-seq	FACS	In vitro transcription	3′ end	Background noise is greatly reduced to minimize sampling bias.	The experimental operation and data processing requirements are high.	(111)
ST-seq	GeoMx DSP	PCR	Whole-genome wide unbiased amplification of mRNA	Retain spatial information, perfectly integrated with organizational morphology (H&E/IF)	The analysis is complex, high costs, complicated sample preparation and experimental procedures	(112)

## 2 Applications of single-cell sequencing technology in hepatic echinococcosis

### 2.1 Analyzing hepatic echinococcal lesion heterogeneity in hepatic echinococcosis

#### 2.1.1 Analyzing hepatic echinococcal lesion heterogeneity in CE

**Figure 3:**
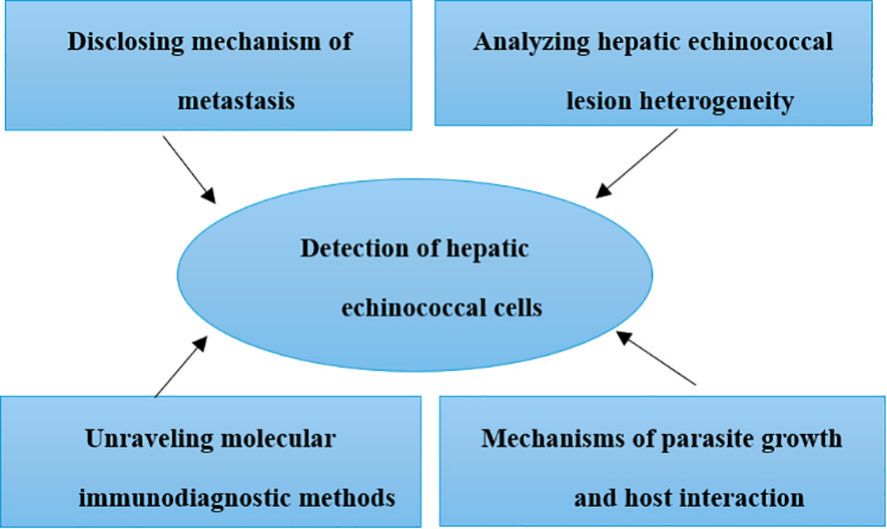
Applications of single cell sequencing technology in detecting echinococcal cells.

CE is a zoonotic disease that has spread worldwide (**Figure 3**). Previous studies have shown that various infiltrating immune cells can accumulate around lesions and form a unique microenvironment (39, 40). However, the cellular composition and heterogeneity of the hepatic CE microenvironment are not fully understood and require further investigation. Some studies have found that Echinococcus granulosus has gradually evolved a set of elaborate and complex mechanisms during the long-term parasitism process that can circumvent the host’s strong immune response and maintain a delicate and sTable long-term balance between the host immune system and growth of the parasite itself. Parasites are pivotal biological models for investigating immune regulation. Their survival hinges on a dynamic equilibrium between host and pathogen, maintained through parasitic strategies of tissue invasion and immune evasion. Host immunity must balance pathogen clearance with preventing excessive self-damage: overactive immunity eradicates parasites, whereas immunosuppression risks host fatality due to unchecked parasitic proliferation. Crucially, disruptions occurring before parasite reproductive maturity threaten pathogen population sustainability. Notably, parasites often exploit host immune components (e.g., regulatory T cells, TGF-β) to complete their life cycles, while host immune responses exhibit dual protective and detrimental roles. For instance, Schistosoma mansoni-induced portal fibrosis and cerebral malaria neuropathology caused by Plasmodium falciparum represent secondary tissue damage stemming from host immune defenses. These pathologies correlate directly with immune response intensity and regulatory patterns, underscoring the intricate coevolutionary interplay in host-parasite relationships (41, 42). Recently, single-cell sequencing has been applied to cancer and immune cells in hepatocellular carcinoma to reveal the dynamic heterogeneity of immune cells in the cancer microenvironment (43) (**Table 3**). In 2021, Aimaiti Yasen’s team (9) performed Single-cell RNA sequencing on CE,revealed heterogeneity in hepatic immune cell infiltration. Analysis of 81,865 immune cells from PB, PL, and AN liver tissues identified 23 distinct subsets with tissue-specific variations. Despite similarities between PL and AN (**Table 1**) immune profiles, PL tissues exhibited elevated proportions of type 2 innate lymphocytes (ILC2) (**Table 1**) and plasmacytoid dendritic cells (pDCs) (**Table 1**), alongside upregulated immunosuppressive gene NFKBIA. Enhanced interactions among CD4+ T cells, ILC2, and pDCs in PL regions promoted an immunosuppressive microenvironment via increased NKG2A/HLA-E checkpoint expression. Experimental validation confirmed higher NFKBIA and NF-κB levels in PL versus AN tissues, underscoring their role in host-parasite immune dynamics (44). The investigators also identified PL tissues exhibited enrichment of regulatory CD4+ T cells and Th2-CD4+ T cells compared to AN tissues. Enhanced interactions among CD4+ T cells, ILC2, and pDCs in PL regions upregulated immunosuppressive checkpoint gene NKG2A/HLA-E while suppressing pro-inflammatory signals, promoting an inhibitory microenvironment in CE lesions. Experimental validation confirmed elevated NFKBIA and NF-κB levels in PL tissues. This suggests NFKBIA suppresses NF-κB signaling to dampen local immune responses, facilitating parasite survival. These findings provide insights into CE pathogenesis and potential therapeutic targets at the cellular level.

**Table 3:** Single-cell transcriptomic sequencing and cellular heterogeneity in hepaticmultilocularis echinococcus.

Study/year	Cellular Heterogeneity	Methods	Key Results	Reference
Yasen A et al 2021.	Heterogeneity In the profile of infiltrating immune cells in the hepatic cystic echinococcosis microenviron-ment.	Single-cell RNA sequencing of PB,PL, and AN samples from 4 patients with cystic echinococcosis.	Twenty-three unique cell subsets were identified, and infiltrating immune cells were found to differ signifi-cantlyacross tissue settings.	(9)
Jiang T et al 2022	To reveal single-cell heterogeneity of liver-infiltrating lymphocytes in chronic AE infection and explore related immune mechanisms.	Single-cell TCR sequencing on CD45+ cells from PB, PL, and AN liver tissues of 4 AE patients.	Significant differences in subset distribution and transcriptome profiles in hepatic and perihepatic microenvironments, proportions of exhausted CD8+ T cells and ILC2 were markedly increased in PL tissues.	(49)
Wang M et al 2024	Analyze the expression characteristics of CD244 on depleted CD8+ T cells in the near liver tissue (CLT) of patients with AE.	(1)Single-cell RNA sequencing; (2)Immunofluorescence; (3)Flow cytometry and in vitro/in vivo models.	CD244+ CD8+ T cells in CLT showed terminally Differentiated phenotypes, with significantly reduced secretion of IFN-γ and TNF-α.	(53)

To explore potential new markers and therapeutic targets, differential gene expression analysis was carried out on ILC2 and pDC in PL and AN tissues, with the aim of revealing the differential characteristics of ILC2 and pDC at the gene expression level. The results revealed that the differentially expressed genes in ILC2 and pDC were closely related to liver failure and fatty liver, indicating that the disease has relatively benign characteristics. The hepatic immune microenvironment exhibits a high degree of heterogeneity across different diseases and a high degree of diversity among different individuals (45). In 2022, Jiang XF et al. (46) provided new insights into the heterogeneity of endothelial and immune cells through their study of the single-cell landscape of CE at different stages. Hydatid cysts and angiogenesis are key features of cystic echinococcosis, and immune and endothelial cells play important roles in disease progression. Cell communication analysis revealed that SPP1(+) (secreted phosphorylated glycoprotein 1) macrophages can interact with endothelial cells and exhibit pro-angiogenic functions. Furthermore, there was a significant association between SPP1 (+) macrophages and endothelial cells using Vegfa-Vegfr1/Vegfr2. In addition, SPP1 (+) macrophages interact with other immune cells through specific ligand-receptor pairs, which may contribute to their immunosuppressive functions. This research conducted a systematic investigation into the ecological system of cystic echinococcosis, and pioneered the identification of macrophages with phase-specific SPP1(+) expression during infection. This crucial discovery facilitates the comprehension of the regulatory rules of angiogenesis, and further reveals the mechanisms underlying immune evasion after pathogen infection (46). AE is a lethal parasitic disease, and human AE occurs mainly in the liver, where AE grows as tumors that often invade and destroy the liver and other organs (47). Due to its aggressive growth, if left promptly untreated, 90% of infected people die within 10 to 15 years of diagnosis (48). Therefore, there is an urgent need to further explore the pathological immune mechanism and the body’s protective mechanism against AE infection to provide a theoretical basis and direction for the development of more effective treatments that can address the shortcomings of existing treatment methods and improve the therapeutic effect of AE and patient prognosis. For supplementary validation of SPP1^+^ macrophages, it will construct a gene knockout mouse model and conducted neutralizing antibody intervention experiments. It will establish a SPP1 knockout mouse model infected with alveolar echinococcosis. The lesion growth rate, fibrosis degree and alterations of immune microenvironment will be compared between knockout mice and wild-type mice. Meanwhile, in vivo intervention experiments with SPP1 neutralizing antibody will be carried out to verify the effects of SPP1 signal blockade on reversing macrophage polarization, improving inflammatory microenvironment and inhibiting parasite proliferation, so as to clarify the causal value of SPP1 as a therapeutic target.

#### 2.1.2 Analyzing hepatic echinococcal lesion heterogeneity in AE

In 2022, Jiang et al. (49) used scRNA-seq and single-cell TCR sequencing (scTCR-seq) to isolate CD45+ cells from PB, PL, and AN liver tissue samples from four patients with AE. Their results revealed the single-cell heterogeneity of liver-infiltrating lymphocytes in individuals with chronic AE infections and provided insights into related immune mechanisms. A total of 83,921 CD45+ lymphocytes were analyzed in the liver tissue of patients with AN, and 23 large cell subsets were identified. Significant differences were observed in the distribution and transcriptomic characteristics of these cell subsets in the liver and perihepatic microenvironments. In particular, the proportion of depleted CD8+ T cells and ILC2 in PL tissue was significantly increased. Among these cell types, CD8+ T cells play a cytotoxic role in immune responses (50). In-depth studies have found that the expression characteristics of these cell subsets are closely related to the inhibition of lesion metastasis and immune response. An analysis of the five CD8+ T cell subsets revealed that only CD8+ mucosa-associated invariant T (MAIT) cells were enriched in PL samples, and TRAV1-2_TRAJ33_TRAC TCRs were amplified. In a study of 11 subsets of CD4+ T cells, Th17 cells and inducible regulatory T cells (iTregs) showed preferential enrichment characteristics in PL samples, and theirhighly expressed genes were closely related to cell invasion behavior, inhibition of AE transfer mechanisms, and inflammatory immune responses. Notably, this information provides a key basis for further investigation of disease-related immune mechanisms. Evidence gathered in these immunosuppressed states suggests that this state contributes significantly to disease progression, leading to the proliferation of AEs beyond the body’s normal immune surveillance into a state of loss of control (51). In this state, AE proliferates and invades to form a growing liver lesion consisting of a population of AE vesicles that bind primarily to the host connective tissue and infiltrating immune cells. Recently, histopathological analysis of human AE liver specimens revealed that the surrounding immune infiltrates consisted of macrophages, lymphocytes, granulocytes, and myofibroblasts. In this sample, subsets of CD4+ and CD8+ T cells were found to accumulate around AE foci in the liver (52). These findings provide a key reference for optimizing immunotherapy strategies for AEs and for formulating disease prevention and control measures. They also clarified the central role of the immune system in maintaining health and defending against parasitic infections.

In 2024, Wang Maolin et al. (53) used various advanced technologies to conduct a comprehensive and in-depth exploration of the immune mechanisms in patients with AE. First, single-cell RNA sequencing technology was used to analyze the expression characteristics of CD244 on depleted CD8+ T cells in the near liver tissue (CLT) (**Table 1**) of patients with AE and accurately determine its genetic expression pattern. Simultaneously, immunohistochemistry and immunofluorescence were used to visualize and localize the expression of CD244 to ensure accurate expression information at the tissue and cell levels. Furthermore, using flow cytometry and in vitro and in vivo models, the effect of CD244 on the differentiation trend and effector function of CD8+ T cells in patients with AE was comprehensively evaluated, and its key role in the regulatory network of immune cell function was thoroughly investigated. To elucidate the mechanism of CD244 from the perspective of cellular energy metabolism, the specific role of CD244 in the mitochondrial function of CD8+ T cells was systematically evaluated by measuring the level of reactive oxygen species (ROS) production and changes in the oxygen consumption rate (OCR) (**Table 1**). It was found that CD244+ and CD8+ T cells in CLT showed terminally differentiated phenotypic characteristics, and the secretion of IFN-γ and TNF-α cytokines was significantly reduced. In vitro experiments showed that CD8+ T cells derived from CD244-deficient mice exhibited a unique ability to produce higher levels of IFN-γ, TNF-α, and granzyme B, suggesting that CD244 deletion may alter the functional status of CD8+ T cells. Further in vivo studies revealed that CD244 deficiency significantly enhanced the ability of CD8+ T cells to secrete IFN-γ and TNF-α, and the direct effect of this enhanced immune cell function effectively inhibited the growth of Echinococcus multilocularis. Notably, this finding provides a potential direction for the use of immunotherapy in the treatment of this disease. In addition, studies at the cellular metabolism level have found that CD244 deficiency triggers changes in liver CD8+ T cells, which are manifested by a decrease in ROS levels and a significant increase in the OCR of adenosine triphosphate (ATP) linkages in cells. This suggests that CD244 may play a key role in regulating the metabolism and function of CD8+ T cells and that CD244 promotes AE disease progression by mediating immune exhaustion in CD8+ T cells. This observation also improves our understanding of the mechanism of action of CD244 in the disease process of AE and opens up a new path for the subsequent development of precision immunotherapy strategies for this disease, with potential directions and targets for future research. According to the above mechanistic exploration of CD244^+^ exhausted CD8^+^ T cells, a series of validation strategies were proposed.Immune intervention verification of CD244^+^ exhausted CD8^+^ T cells: CD244 blocking antibody will be used for in vivo intervention. It will explore whether blocking CD244 signal can reverse the exhaustion state of CD8^+^ T cells, restore their cytotoxic function, and activate the host immune response against echinococcosis, thereby evaluating the potential of CD244 as an immune checkpoint therapeutic target.

### 2.2 Disclosing mechanism of metastasis

AE are “malignant parasitic diseases” characterized by chronic, progressive, and infiltrative growth (1, 54). Echinococcus multilocularis eggs can enter the duodenum if accidentally ingested, and subsequently, more than 70% of the ingested Echinococcus multilocularis eggs enter the portal vein system and remain in the liver, eventually forming an AEs. AEs can affect the entire liver, disrupt its anatomy, and impair liver function. Pharmacological treatment of AEs mainly involves benzimidazoles, albendazole, and mebendazole; however, the treatment cycle is long. Without effective intervention, the condition progressively worsens, and most patients develop obstructive jaundice when the lesion expands and erodes the biliary tract. If a secondary infection develops in the liquefaction cavity, an abscess may also develop. If large lesions erode most of the liver tissue, they may also cause portal hypertension and liver dysfunction, and patients may eventually die from liver failure, biliary system infection, and metastasis to the lungs, brain, and other organs (55).Hepatic AE can metastasize to the lungs, brain, kidneys, and surrounding tissues through invasion,implant metastasis, and hematogenous and lymphatic routes. However, there is a relative lack of research on hepatic AE lymph node metastasis, and conclusive evidence for regional lymphatic metastases remains lacking (56, 57). Lymph node metastases may occur because AE utilizes the deep and superficial multidirectional lymphatic reflux pathways in the liver to drain regional lymph nodes (58). Although several related studies have been reported, there is still a lack of broader and more systematic literature in the field of lymph node metastasis that is needed to clarify the standard rules for diagnosis and treatment (59–62).

In 2022, Jiang et al. (49) used single-cell RNA and TCR sequencing to analyze samples obtained from four patients with advanced AEs in a study on the single-cell heterogeneity of liver-infiltrating lymphocytes in individuals infected with chronic Echinococcus multilocularis. A total of 83,921 CD45 lymphocytes were isolated from the PB, PL, and AN liver tissues. After careful analysis and identification, 23 large cell subsets were identified. There were significant differences in the distribution of these cell subsets in the liver and peripheral blood, as well as in the transcriptome characteristics. The proportions of depleted CD8+ T cells and ILC2 cells in PL tissues showed a significant increasing trend. Furthermore, in a study of five different CD8+ T cell populations, a unique phenomenon was observed: only CD8+ mucosa-associated invariant T (MAIT) cells were significantly enriched in PL samples, and TRAV1-2_TRAJ33_TRAC TCR also showed clonal expansion. When analyzing the 11 subsets of CD4+ T cells, Th17 cells, and induced regulatory T cells (iTregs), preferential aggregation was observed in PL samples. An in-depth study of these cells revealed that their highly expressed genes play an important role in cell invasion, lesion metastasis, and inhibition of the inflammatory immune response (49).

### 2.3 Unraveling molecular immunodiagnostic methods

#### 2.3.1 Application of diagnostic techniques in Echinococcosis

The clinical manifestations of echinococcosis are often nonspecific, and most patients do not show symptoms until the disease progresses to an advanced stage of the disease. This feature makes the diagnosis of the disease difficult, particularly in its early stages. Among the many diagnostic methods, ultrasound imaging has become the most commonly used in clinical practice because of its economic advantages and rapid diagnosis. In addition, high-resolution imaging techniques, such as computed tomography (CT) and magnetic resonance imaging (MRI), are widely used to detect lesions and atypical echinococcosis at specific anatomical sites (63). A significant limitation of these imaging techniques is that echinococcal cysts cannot be accurately distinguished from other type of cysts. Moreover, imaging analysis often relies on the presence of relatively large cysts in patients with CE in the advanced stages of the disease, which limits the possibility of early diagnosis (13). Serological testing may be superior to imaging techniques in terms of diagnostic timeliness and can identify diseases earlier than other methods (13). Serological testing can be used as an adjunct diagnostic tool; however, its limitations include potential cross-reactivity and the inability to distinguish between current and past infections (64–66). In addition, it is difficult to identify small cystic lesions using imaging techniques. Echinococcosis cysts are not easily distinguished from cysts caused by other conditions, such as liver abscesses, Caroli disease, bile duct cysts, and cystadenomas (63, 67–69). The long incubation period and complex clinical manifestations of this disease make clinical diagnosis difficult. Prompt imaging and serological testing are recommended for symptomatic patients, and clinical findings alone have limited diagnostic utility (70). Several immunological methods have been successfully developed (71). However, the sensitivity and specificity of these immunological assays have not been established and can fluctuate depending on the testing environment, sample characteristics, and other factors, particularly in the case of CE testing (72–79). The molecular diagnostic methods for AE and CE mainly include serological detection (78), antigen detection, cellular immunoassays, PCR molecular diagnosis, and novel biomarker detection. Serological tests mainly include enzyme-linked immunosorbent assay (ELISA) (72), immunoblotting (80), indirect hemagglutination test (IHA) (81), and immunofluorescence antibody test (IFAT) methodologies. Given the limitations of existing diagnostic tools, the detection of cell-free DNA released by Echinococcus in peripheral circulation using high-throughput sequencing is expected to provide a potential novel etiological biomarker. In routine screening, AE and CE can be detected using similar antibody-based methods such as ELISA, because both parasites share common antigenic epitopes, and the assays both aim to detect specific antibodies against Echinococcus.

#### 2.3.2 Application of single-cell sequencing in the diagnosis of CE

In 2020, Ji J et al. (82) conducted a systematic analysis of cell-free Echinococcus DNA in the plasma of patients with echinococcosis using ultra-high-throughput sequencing technology (i.e., single-cell sequencing technology). Their study confirmed the potential application value of this biomarker in the clinical diagnosis of echinococcosis.Notably, the findings confirmed the potential application value of this biomarker in the clinical diagnosis of echinococcosis. Plasma samples were collected from 23 patients with echinococcosis to explore the molecular characteristics of the disease. Total cfDNA was first extracted from the collected plasma samples and sequenced using a high-throughput sequencing platform. After sequencing, an average of 282 million read pairs were obtained per plasma sample. To further investigate the key information in the sequencing data, a bioinformatics workflow was used to analyze the sequencing data in combination with the Echinococcus sequence database. After the successful identification of acellular Echinococcus readings, the study found that these readings accounted for a range of 1.8e-5 to 4.0e-9 of the total clean readings. Further comparison of the fragment length distribution of cfDNA between Echinococcus and humans showed that the length range of free Echinococcus DNA in CE was broad. In contrast, the free Echinococcus DNA of AE showed a significant peak at approximately 135 bp. Furthermore, the study found that the majority of acellular Echinococcus DNA readouts originated from a uniformly distributed nuclear genome, a phenomenon that may suggest a random release pattern of acellular Echinococcus DNA Ultrahigh-throughput sequencing was used to analyze the concentration of free Echinococcus DNA in the plasma, fragment length, and release source of echinococcosis in the plasma of patients with the disease. A better understanding of the characteristics of free Echinococcus DNA in the plasma may provide support for its future application as a diagnostic biomarker.

Presently, the detection method has been validated by multicenter clinical trials and has successfully confirmed its application prospect of transformation from basic research to clinical diagnostic technology. Cell-free DNA released into peripheral circulation by Echinococcus tapeworms is a potential diagnostic biomarker of Echinococcosis. Cell-free DNA testing has been successfully applied for the detection of a variety of parasitic diseases, including Plasmodium infection, trypanosomiasis, and leishmaniasis (83). Cell-free DNA is composed of nucleic acid fragments released from extracellular cells or produced by apoptosis/necrosis and is mainly distributed in body fluids, such as plasma, urine, and cerebrospinal fluid (84). Its presence in plasma and serum has been studied, and it can be stably detected and quantified by high sensitivity PCR techniques, such as qPCR or ddPCR (85–87). In 2020, Wan Z et al. (88) sequenced cfDNA in the plasma of patients with echinococcosis and confirmed the presence of Echinococcus DNA in the patient samples. To improve the sensitivity of this assay, They developed an innovative method based on repetitive, region-targeted next-generation sequencing. Simulation results showed that the targeted sequencing technology was extremely sensitive, and only 0.1% of the echinococcal genome could be detected in 1 mL of plasma. The method performed well in the actual detection of patient plasma samples, with an area under the ROC curve (AUC) of 0.862, detection sensitivity of 62.50%, specificity up to 100%, and a corresponding Youden index of 0.625. This study provides strong evidence for utilizing cfDNA fragments released from plasma by Echinococcus spp for diagnosis.The highly specific detection of echinococcal infections has been achieved using repetitive region-targeted sequencing methods. This achievement opens up a new path for potential non-invasive screening and diagnosis of echinococcosis and is expected to promote the development and innovation of related medical testing technologies, thus bringing more convenient and efficient diagnosis and treatment solutions to patients. Rapid advances in high-throughput sequencing technology have made it possible to sequence cfDNA in both research and medical settings. Compared with target-based PCR, sequencing can provide more comprehensive cfDNA information. The detection of free echinococcal DNA in the plasma of patients with echinococcosis using single-cell sequencing technology has high sensitivity and specificity, but its limitations are obvious. The content of parasite DNA in plasma is very low, it is susceptible to host DNA interference, requires deep sequencing and bioinformatics optimization, has a high cost, the data analysis is complex, it is difficult to meet the needs of a rapid clinical diagnosis, and there is no unified detection process,threshold criteria and clinical validation guidelines.

At present, the diagnosis of echinococcosis relies on imaging (ultrasound, CT, MRI), which is the gold standard for locating the lesion. Serologic tests (ELISA, Western blotting) detect specific antibodies; however, there is cross-reactivity with other parasites. Targeted PCR is sensitive to specific parasite mitochondrial genes but requires pre-targeting of the pathogen. And there are various differences in its limitations and advantages. For the potential diagnostic biomarker cfDNA, it conduct a prospective clinical cohort study, enrolling patients with hepatic echinococcosis at different disease stages, healthy populations and patients with other liver diseases as controls. For cfDNA, a biomarker with promising diagnostic potential, it can plan to launch a prospective multicenter clinical observational study.This study will consecutively enroll hepatic echinococcosis patients at different disease stages, including newly diagnosed cases, active cases and post-operative follow-up cases. Meanwhile, healthy individuals and patients with other benign or malignant liver diseases such as viral hepatitis, liver cirrhosis and liver tumors will be enrolled as control groups.Peripheral blood plasma samples of all participants will be collected in a standardized manner for cfDNA extraction, library construction and sequencing analysis. Corresponding clinical imaging indicators, laboratory biochemical parameters and long-term follow-up data will be recorded simultaneously. By comparing the differences in cfDNA concentration and fragment characteristics among different groups, They aim to systematically evaluate the diagnostic performance of cfDNA in the early differential diagnosis, dynamic monitoring of disease activity, early warning of postoperative recurrence and prognostic assessment of hepatic echinococcosis. Related indicators including sensitivity, specificity, positive predictive value and negative predictive value will be calculated to construct a targeted clinical diagnostic prediction model.This prospective clinical study is expected to clarify the clinical application value of cfDNA in the diagnosis and treatment of hepatic echinococcosis. It will fill the gap that current relevant researches are merely limited to basic correlation analysis, and promote the transformation of cfDNA from an experimental biomarker into a non-invasive and convenient auxiliary tool for clinical screening and long-term monitoring, providing novel evidence for the precise diagnosis and treatment of hepatic echinococcosis (**Table 4**).

**Table 4:** Advantages and limitations of molecular immunodiagnostic methods for echinococcosis.

Study/year	Molecular-Immuno-Diagnostic methods	Merits	limitations	Reference
Kalantari E et al., 2010.	**1. Serologic testing (1):** Enzyme-Linked immuno-sorbent assay (ELISA).	High sensitivity for large-scale screening.	Cross-reactivity with other parasitic infection.	(72)
Perruzzu A et al., 2022.	**1. Serologic testing (2):** Immuno-Oblotting.	High specificity	The operation is complex and time-consuming.	(113)
Auer H et al., 1988.	**1. Serologic testing (3):** Indirect Hemagglutination test (IHA).	The operation is simple and the cost is low.	Sensitivity and specificity are low.	(81)
Kronenberg PA and Zhang WB et al., 2012.	**1. Serologic Testing (4):** Immuno-Fluorescence Antibody test (IFAT).	Intuitive.	A fluorescence microscope is required, and the operation is complicated.	(71, 114)
Kronenberg PA et al., 2022.	**2. Antigen tests.**	A direct reflection of the presence of pathogens	Sensitivity is low, especially in early infection or when parasite load is low.	(115)
Kellar KL et al., 2003.	**3. Cellular (cytokines) Immuno-assays.**	It can reflect the immune status of the body.	The operation is complex and the degree of standardization is low.	(116)
Knapp J et al., 2023.	**4. PCR Molecular Diagnostics.**	It has high specificity and is able to distinguish between different species.	Specialized equipment and technology are required, and the cost is higher.	(117)
Ji J et al., 2020	**5. High-throughput sequencing to detect cell-free DNA released by *Echinococcus* in circulation.**	It is highly sensitive and can reflect the activity status of pathogens.	The technology is still immature, and the clinical application is limited.	(82)

#### 2.3.3 Application of single-cell sequencing in the diagnosis of AE

In recent years, single-cell RNA sequencing and spatial transcriptomics technologies have been widely adopted to identify novel diagnostic biomarkers and screen target antigens for diagnostic antibodies against alveolar echinococcosis (AE), establishing a dual research framework consisting of stratified diagnosis via host immune biomarkers and precise detection using parasite-derived antigen-antibody pairs.In studies on host-derived diagnostic biomarkers, multiple high-resolution omics investigations have delineated distinct immune cell and molecular expression signatures within AE lesions. A spatiotemporal transcriptomic study published in 2025 confirmed that Spp1^+^ monocyte-derived macrophages constitute a lesion-specific cell subset highly enriched in AE lesions. These cells are barely detecTable in healthy liver tissue and show no prominent accumulation in early cystic echinococcosis (CE) lesions. The in situ spatial distribution pattern of this cell subset facilitates the identification of tiny, occult early AE lesions and differentiates active AE lesions from normal hepatic tissue. Nevertheless, SPP1 acts as a universal marker of host injury and profibrotic response and lacks absolute disease specificity for AE. Its upregulation can be triggered by a spectrum of hepatic disorders, including hepatocellular carcinoma, liver fibrosis, and advanced CE. Thus, SPP1 can only serve as an auxiliary discriminative marker for in situ tissue examination and cannot be used alone for clinical differential diagnosis or serological testing of AE. Beyond macrophage-related biomarkers, single-cell RNA sequencing on clinical liver specimens from AE patients has verified that lesion-specific exhausted CD8^+^ T cells, Th2 cells, and their signature gene panels (e.g., Btg1, Nr4a1) are strongly correlated with lesion invasiveness and disease activity. These signatures enable the construction of non-invasive stratified diagnostic models and postoperative recurrence monitoring systems, which address the limitations of conventional Em18 serological assays, namely false-positive results and inability to assess disease activity (89, 90). For the development of specific diagnostic reagents, single-cell omics enables high-throughput screening of conserved, parasite-specific antigens of Echinococcus multilocularis, making it a pivotal preliminary technique for developing novel AE diagnostic antibodies. Current research has identified highly specific parasite membrane antigens such as P29 and EmCRT through single-cell immune profiling, and successfully generated monoclonal and polyclonal antibodies that reliably eliminate cross-reactivity between AE and CE. ELISA platforms and dual-modal imaging probes constructed with these antibodies exhibit superior sensitivity for detecting minute early AE lesions compared with classic Em18 diagnostic kits, offering novel targets and translational strategies for high-specificity serodiagnosis and early imaging diagnosis of AE (91–93).

In conclusion, host immune biomarkers identified via single-cell profiling are mainly applied to AE disease stratification and lesion activity evaluation yet possess limited specificity. In contrast, parasite-derived antigens and matched antibodies screened using single-cell technologies demonstrate high specificity for AE, representing the core direction for the development of precise AE diagnostic kits in the future.

### 2.4 Mechanisms of Echinococcus growth and host interaction

In recent years, researchers have used single-cell sequencing to construct Echinococcal cell atlases, and for the first time, the cellular heterogeneity of the protoscoleces and germinal layers of Echinococcus. Researchers have found that specific stem cell-like subpopulations may be involved in parasite regeneration and vesicle formation. During Echinococcus development, the unique cell proliferation pattern relies on an undifferentiated core group of germinal cells. These cells resemble the pluripotent neonatal cells found in free-living flatworms, such as planarians, and are thought to bethe only cells with a sustained proliferative capacity throughout the parasite’s lifecycle.In the larval stage of Echinococcus multilocularis, the developmental process exhibits remarkable adaptability. The larvae grow aggressively in vesicle-like structures within the tissues of intermediate hosts (e.g., rodents or humans) and continue to produce a large number of protoscoleces through asexual budding reproduction. This near-limitless proliferative potential suggests that germinal cells have stem cell-like properties, including totipotent differentiation (generating all larval cell types) and long-term self-renewal to sustain parasite proliferation in the host (94).

#### 2.4.1 Application of sc-seq in exploring growth and interaction mechanisms of AE

To date, single−cell sequencing has been applied to investigate the growth and development of Echinococcus multilocularis (AE), while similar studies in Echinococcus granulosus (CE) have not yet been conducted. In 2022,Xf Nian et al. (95) Based on high-throughput transcriptome sequencing and microarray technology, the dynamic expression changes of lncRNA and mRNA in mouse liver during AE infection were revealed, and 31 mRNAs and 68 lncRNAs were found to be persistently dysregulated throughout the infection timeline.The growth, proliferation, and survival of multilocularis Echinococcus in the liver of intermediate hosts are highly dependent on the complex regulation of host immune responses.,which revealed that lncRNAs play a potential regulatory role in regulating the differentiation of Th cell subsets in AE hosts and Em infection specifically activates two innate immune pathways, Toll-like receptor and RIG-I-like receptor, at 30 dpi, and the process of Em infection is accompanied by a dynamic imbalance of host Th1/Th2/Th17 immune responses.Transcriptomic studies can help uncover the potential function of regulatory RNA molecules, particularly long non-coding RNAs (lncRNAs), in manipulating host immune responses for parasite survival. As an important class of regulatory molecules, lncRNAs have been shown to play a key role in the regulation of host-pathogen interactions in a variety of infections, including viruses, bacteria, and parasites. In 2024, Herz et al. (96) used Illumina high-throughput sequencing technology to perform in-depth sequencing analysis of complementary deoxyribonucleic acid (cDNA) from echinococcal vesicles, germinal cell knockout tissues, and echinococcal primary cell cultures. After rigorous bioinformatics analysis, a set of approximately 1180 genes was successfully identified, all of which were closely related to Echinococcus germinal cells. This group of genes not only includes a large number of known stem cell markers but also contains many genes that play an important role in key biological processes, such as cell replication, cell cycle regulation, mitosis, meiosis, epigenetic modification, and nucleotide metabolism. In particular, 44 transcription factors associated with stem cells were identified. According to existing studies and bioinformatics predictions, these transcription factors are likely to be involved in the differentiation and regulation of germinal cells, which is of great significance for maintaining germinal cell pluripotency.Through in situ-hybridization and pulse-chase experiments, a noteworthy discovery was made, namely, the identification of a new and versatile echinococcal stem cell marker, EmCIP2Ah. Simultaneously, sufficient experimental evidence was collected to confirm the existence of a special subset of slow-circulating stem cells capable of expressing the extracellular matrix factor, Emkal1. RNA sequencing analysis of primary cell cultures showed that stem cells derived from Echinococcus cells exhibited a strong differentiation ability. They were not only capable of differentiating into a variety of cell types specific to Echinococcus, but were also able to further differentiate into cells expressing genes specific to protoscolex, adult, and oncosphere. It is worth mentioning that the gene set also includes EmTRPMPZQ, a homolog of the schistosomiasis praziquantel target. Finally, it was experimentally determined that a specialized cell subset of primary cell cultures is capable of expressing tumor necrosis factor α. Further studies have found that the addition of mammalian tumor necrosis factor α can significantly accelerate the development of germinal cells to echinococcal vesicles.

In summary, this study conducted a comprehensive and in-depth exploration of the transcriptome of echinococcal germinal cells using multidimensional experimental and analytical methods.These findings not only confirm the strong differentiation ability of stem cells derived from Echinococcus but also highlight the great potential of primary germinal cell cultures for studying the development of this parasite. These results have important theoretical value for the in-depth exploration of the mechanism of action of echinococcal stem cells in the process of parasite development and provide key data support and a theoretical basis for the future design and development of antiparasitic drugs that can specifically act on parasite germinal cells.

## 3 Combined application of single-cell sequencing and spatial transcriptomics in Echinococcosis

Single-cell RNA sequencing accurately identifies cell identity, subtypes and molecular features in hepatic echinococcosis lesions (97). yet suffers from loss of spatial information, difficult dissociation of liver and cyst wall tissue, cell shedding, low capture of rare cells and obvious batch effects. Spatial transcriptomics preserves intact tissue structure and spatial location, but lacks single-cell resolution and fine cell typing capacity, showing high complementarity in cell identification and spatial analysis. Integrating the two approaches by first performing cell clustering and annotation via scRNA-seq, then mapping annotated cell signatures to spatial transcriptomic loci effectively improves research systematicity and spatial resolution. This strategy precisely depicts spatial distribution of cell subsets, and further compares the local immune microenvironment between cystic and alveolar echinococcosis, cystic echinococcosis possesses intact fibrous cyst wall with zonated immune distribution and immune-suppressive microenvironment facilitating parasite immune escape; alveolar echinococcosis presents invasive blurred lesions, showing gradual gradients of chronic inflammation, immune disorder and progressive fibrosis from lesion center to periphery, providing high-dimensional evidence for revealing microenvironmental heterogeneity, pathological progression and immune escape mechanism of hepatic echinococcosis (98).

In 2025, Zhihua Ou et al. used female BALB/c (Bagg Albino/c) mice aged 6 to 8 weeks in experimental animals. Echinococcus multilocularis protoscoleces were isolated from naturally infected Lasiopodomys fuscus in Yushu, Qinghai Province, and maintained by intraperitoneal passage in mice. After viability identification and quantification, a total of 2000 protoscoleces were inoculated into mice via combined hepatic capsular and intraperitoneal injection. Liver tissues were collected at multiple time points of 4, 8, 15, 37 and 79 days post infection (dpi), with blank negative controls established simultaneously. The harvested liver tissues were embedded in OCT (Optimal Cutting Temperature) and preserved at low temperature, thereby constructing an animal model covering different infection courses.A series of multi-omics sequencing and histological verification were subsequently performed. RNA quality control was conducted on OCT-embedded samples, and only samples with an RNA Integrity Number (RIN) ≥ 7 were adopted for subsequent experiments. Combined with Hematoxylin and Eosin (H&E) staining, samples containing infectious lesions and adjacent host tissues were screened for Stereo-seq. The construction and sequencing of Stereo-seq libraries were completed on the Spatial Temporal Omics platform, following standard procedures including tissue section mounting, methanol fixation, permeabilization, in situ mRNA hybridization, reverse transcription, cDNA amplification and on-machine sequencing. Parallel H&E staining of adjacent tissue sections was performed for pathological evaluation. Matched tissue samples were subjected to bulk RNA sequencing, involving total RNA extraction, concentration detection, library construction and high-throughput sequencing to obtain the global transcriptional profiles of tissues. Immunohistochemistry (IHC) staining targeting MPO and IL-1β was applied to localize neutrophils and characterize inflammatory expression within lesions. The perilesional and distal lesion liver regions were precisely delineated, single-cell suspensions were prepared, and scRNA-seq libraries were constructed using the 10×Genomics platform to achieve high-resolution transcriptomic profiling of hepatic cells. A standardized multi-omics collaborative analytical pipeline was established for data processing. Raw Stereo-seq data were automatically processed via the BGI STOmics (Beijing Genomics Institute Spatial Temporal Omics) analysis pipeline, including barcode demultiplexing, adapter trimming, genome alignment, deduplication and gene quantification. A hybrid reference genome integrating the mouse genome –GRCm39 (Genome Reference Consortium Mouse Build 39) and the Echinococcus multilocularis genome- WBPS15 (WormBase ParaSite release 15) was applied. Background noise was eliminated, and the bin100 unit was adopted for downstream analysis. The Seurat package was utilized for data normalization, dimensionality reduction, clustering and spatial visualization. Combined with H&E pathological images, the distribution of parasite genes and the scope of infectious foci were determined. Cell type deconvolution was performed to dissect the cellular composition of each spatial unit, and GSVA(Gene Set Variation Analysis), gene signature scoring, Pearson correlation analysis and CellChat (Cellular Communication)-based ligand-receptor interaction analysis were conducted to explore immune functional status, cellular colocalization and intercellular crosstalk across distinct spatial regions. For scRNA-seq data, Cell Ranger was used for barcode processing and gene expression matrix generation. Data filtering, doublet removal and integration were implemented in Seurat. Cell clustering, cell type annotation and differentially expressed gene screening were carried out, alongside GO (Gene Ontology) functional enrichment, gene signature evaluation, NicheNet (Niche Network)-mediated ligand regulatory network analysis and SCENIC (Single-Cell Regulatory Network Inference and Clustering)-based transcription factor profiling, to further unravel the functional differentiation and molecular regulatory mechanisms of immune cell subsets. Bulk RNA-seq data underwent quality filtering, sequence alignment and expression normalization. Principal component analysis (PCA) was performed to characterize the overall transcriptional traits, and CIBERSORTx (Cell-type Identification By Estimating Relative Subsets Of RNA Transcripts) deconvolution was adopted to dissect immune cell composition, bridging macroscopic transcriptional alterations and microscopic cellular heterogeneity (90).

Compared with the application of a single sequencing technology, the integrated strategy combining scRNA-seq, Stereo-seq and bulk RNA-seq presents prominent technical superiorities. As an unbiased approach, scRNA-seq enables the comprehensive identification of hepatic cell subsets and their transcriptional characteristics, accurately defining the molecular phenotypes of key effector cells such as neutrophils, Spp1^+^ macrophages and fibroblasts, whereas it is incapable of retaining the spatial location information of cells. In contrast, Stereo-seq can in situ depict the transcriptional discrepancies among parasitic lesions, immune infiltration areas, fibrotic regions and normal hepatic tissues, and dynamically visualize the spatial patterns of immune cell infiltration, parasite-host interaction and fibrotic progression, compensating for the deficiency of insufficient cellular resolution in scRNA-seq. Bulk RNA-seq reflects the holistic gene expression changes of tissues and validates the temporal transcriptional features of the disease, and its deconvolution analysis links bulk phenotypic alterations to alterations in cellular components (9, 49, 90).The synergistic integration of the three technologies enables a multi-dimensional interpretation of the mechanisms underlying immune suppression, inflammatory imbalance and pathological fibrosis in hepatic alveolar echinococcosis from the perspectives of molecular cytology, spatial distribution and temporal dynamics. It also accurately identifies core cellular interaction hotspots and regulatory pathways in the lesion microenvironment. Meanwhile, mutual verification among multi-omics data enhances the reliability of research conclusions and avoids the one-sided understanding of disease mechanisms caused by single-technology research. This study not only provides comprehensive and solid theoretical evidence for screening spatiotemporal specific diagnostic biomarkers and developing targeted therapeutic strategies, but also establishes a reliable multi-omics integrated technical paradigm for exploring the pathogenesis of parasitic infectious disease.

## Discussion

This review focuses on formulating the next research strategy by studying the latest advances in single-cell RNA sequencing in echinococcosis, to discover new biological targets and contribute to the molecular targeted therapy of this disease.Echinococcosis is a zoonotic disease caused by larval infection with the Echinococcus tapeworm. Its pathogenic mechanism is complex and involves long-term interplay between the host immune response and parasite escape. In recent years, single-cell sequencing technology has been widely used in echinococcosis research to elucidate the heterogeneity of immune cells in the host immune microenvironment, thereby revealing the subpopulation differentiation and functional status of host immune cells (macrophages, T cells, B cells, etc.) in the infection site (such as liver) The immunosuppressive mechanism was systematically analyzed, and through evaluation of the dynamic changes in regulatory T cells and myeloid suppressor cells in the infected host, it was determined that echinococcosis echinococciosis may reshape the immune microenvironment and inhibit the Th1/Th17 immune response by secreting exosomes or specific metabolites. A parasite cell atlas was constructed, and the cellular heterogeneity of the protoscoleces and germinal layers of Echinococcus was analyzed for the first time (9, 49). Specific stem cell-like subsets were identified to be involved in parasite regeneration and vesicle formation. By comparing transcriptome differences between infected patients and healthy human peripheral blood mononuclear cells, it was observed that the expression levels of specific chemokines in CD8+ T cells and free echinococcal DNA in the peripheral circulation could be used as new potential diagnostic markers (82). Explore new drug targets, Identifying parasite germinal layer-specific highly expressed genes as novel drug targets, combined with CRISPR screening to validate chemotherapeutic potential, offers innovative strategies for echino-coccosis mechanism research and therapy development. These studies provide some reference data for the next steps of molecular target screening and validation experiments.Single-cell RNA sequencing (scRNA-seq) has outstanding advantages: it resolves cellular heterogeneity, avoids the averaging bias of bulk RNA-seq, constructs high-resolution single-cell transcriptomic profiles, reconstructs cell differentiation and pathological trajectories by pseudotime analysis (99). It precisely characterizes the immune microenvironment, and adapts to small clinical samples, enabling in-depth exploration of cell subtypes, gene expression and host-parasite interaction in hepatic echinococcosis.Nevertheless, scRNA-seq presents systematic limitations in this disease research. The dense structure of liver tissue and cyst wall, enriched with fibrosis, calcification and mucus matrix, brings great difficulty to tissue dissociation, easily causing cell damage, apoptosis and low cell viability (14). Severe cell shedding during dissociation leads to loss of in situ cell components, while the capture efficiency of rare cell subsets is poor, failing to fully enrich key rare immune and lesion cells. Obvious batch effects derived from samples, reagents and sequencing platforms introduce systematic bias and reduce data reproducibility. Moreover, the severe imbalance of host and parasite RNA makes dominant host transcripts mask low-abundance parasitic RNA, resulting in inaccurate genome alignment and species annotation. Inherent technical flaws include limited gene capture efficiency, frequent dropout events of low-abundance genes, amplification noise, high cost, and only 3-end sequencing that cannot analyze full-length transcripts (100).

In addition, tissue dissociation completely loses spatial information and ignores the strong spatial heterogeneity of lesions; scRNA-seq mainly focuses on mRNA and lacks coverage of non-coding RNA and epigenetic regulation, only providing static transcriptional snapshots rather than dynamic cell crosstalk. Meanwhile, the lack of mature cell lines and organoid models hinders the functional verification of key genes and cell subsets screened by sequencing. In summary, although scRNA-seq provides high-resolution support for studying cellular heterogeneity, immune microenvironment and host-parasite interaction in hepatic echinococcosis, it is restricted by ineviTable problems such as difficult dissociation of liver and cyst wall tissue, cell shedding, low capture of rare cell subsets, batch effects, and host-parasite RNA imbalance, as well as technical and interpretative bottlenecks. Combining spatial transcriptomics, multi-omics strategies, optimized dissociation protocols and animal models is required to compensate for these deficiencies and further elucidate pathogenesis and identify diagnostic and therapeutic targets of hepatic echinococcosis.

## Conclusion and outlook

At present,Single-cell sequencing is advancing echinococcosis research to cellular and molecular levels, holding promise for breakthroughs in pathogenesis, precision medicine, and vaccine development through technological innovation and interdisciplinary collaboration in the future. However, This technique has some limitations,challenges persist in sample preparation, data analysis, and clinical translation. In the future, we can rely on newer and more advantageous single-cell full-length sequencing and spatial transcriptomic sequencing to advance the next step of research. Addressing these gaps will require strengthened global cooperation and open-access resource sharing to accelerate therapeutic advancements.

## References

[1] McManus DP Zhang W Li J Bartley PB . Echinococcosis. Lancet. (2003) 362:1295–304. doi: 10.1016/b978-0-12-801238-3.03103-2 14575976

[2] Jun-Jie C . Epidemiological studies on cystic echinococcosis in China─a review. BioMed Environ Sci. (1995) 8(2):122–36. 7546341

[3] Zhenghuan W Xiaoming W Xiaoqing L . Echinococcosis in China, a review of the epidemiology of Echinococcus spp. EcoHealth. (2008) 5:115–26. doi: 10.1007/s10393-008-0174-0 18787915

[4] Czermak BV Akhan O Hiemetzberger R Zelger B Vogel W Jaschke W . Echinococcosis of the liver. Abdom Imaging. (2008) 33:133–43. doi: 10.1007/s00261-007-9331-0 17912581

[5] Liang JL Cai WS Sun ZS . Single-cell sequencing technologies: current and future. J Genet Genomics. (2014) 41:513–28. doi: 10.1016/j.jgg.2014.09.005 25438696

[6] Luecken MD Theis FJ . Current best practices in single-cell RNA-seq analysis: a tutorial. Mol Syst Biol. (2019) 15:e8746. doi: 10.15252/msb.20188746 31217225 PMC6582955

[7] Tirosh I Izar B Prakadan SM Wadsworth MH 2nd Treacy D . Dissecting the multicellular ecosystem of metastatic melanoma by single-cell RNA-seq. Science. (2016) 352:189–96. doi: 10.1126/science.aad0501 27124452 PMC4944528

[8] Zheng C Zheng L Yoo JK Guo H Zhang Y Guo X . Landscape of infiltrating T cells in liver cancer revealed by single-cell sequencing. Cell. (2017) 169:1342–1356.e16. doi: 10.1016/j.cell.2017.05.035 28622514

[9] Yasen A Sun W Aini A Aji T Shao Y Wang H . Single-cell RNA sequencing reveals the heterogeneity of infiltrating immune cell profiles in the hepatic cystic echinococcosis microenvironment. Infect Immun. (2021) 89:e0029721. doi: 10.1128/iai.00297-21 34491790 PMC8594604

[10] Han A Glanville J Hansmann L Davis MM . Linking T-cell receptor sequence to functional phenotype at the single-cell level. Nat Biotechnol. (2014) 32:684–92. doi: 10.1038/nbt.2938 24952902 PMC4337815

[11] P MD J GD Wenbao Z Yurong Y . Diagnosis, treatment, and management of echinococcosis. BMJ (Clinical Res ed). (2012) 344:e3866. doi: 10.1136/bmj.e3866 22689886

[12] Craig PS Larrieu E . Control of cystic echinococcosis/hydatidosis: 1863-2002. Adv Parasitol. (2006) 61:443–508. doi: 10.1016/s0065-308x(05)61011-1 16735171

[13] Craig PS McManus DP Lightowlers MW Chabalgoity JA Garcia HH Gavidia CM . Prevention and control of cystic echinococcosis. Lancet Infect Dis. (2007) 7:385–94. doi: 10.1016/s1473-3099(07)70134-2 17521591

[14] Wen H Vuitton L Tuxun T Li J Vuitton DA Zhang W . Echinococcosis: advances in the 21st century. Clin Microbiol Rev. (2019) 32(2):e00075–18. doi: 10.1128/cmr.00075-18 30760475 PMC6431127

[15] Schweiger A Ammann RW Candinas D Clavien PA Eckert J Gottstein B . Human alveolar echinococcosis after fox population increase, Switzerland. Emerg Infect Dis. (2007) 13:878–82. doi: 10.3201/eid1306.061074 17553227 PMC2792858

[16] Qucuo N Wu G He R Quzhen D Zhuoga C Deji S . Knowledge, attitudes and practices regarding echinococcosis in Xizang Autonomous Region, China. BMC Public Health. (2020) 20:483. doi: 10.1186/s12889-020-8314-8 32293375 PMC7158018

[17] Budke CM Deplazes P Torgerson PR . Global socioeconomic impact of cystic echinococcosis. Emerg Infect Dis. (2006) 12:296–303. doi: 10.3201/eid1202.050499 16494758 PMC3373106

[18] Fu MH Wang X Han S Guan YY Bergquist R Abcde WPW . Advances in research on echinococcoses epidemiology in China. Acta Trop. (2021) 219:21. doi: 10.1016/j.actatropica.2021.105921 33878307

[19] Restrepo AMC Yang YR McManus DP Gray DJ Giraudoux P Barnes TS . The landscape epidemiology of echinococcoses. Infect Dis Poverty. (2016) 5:13. doi: 10.1186/s40249-016-0109-x 26895758 PMC4759770

[20] Moro P Schantz PM . Echinococcosis: a review. Int J Infect Dis. (2009) 13:125–33. doi: 10.1016/j.ijid.2008.03.037 18938096

[21] Eckert J Deplazes P . Biological, epidemiological, and clinical aspects of echinococcosis, a zoonosis of increasing concern. Clin Microbiol Rev. (2004) 17:107–+. doi: 10.1128/cmr.17.1.107-135.2004 14726458 PMC321468

[22] Nakao M Lavikainen A Yanagida T Ito A . Phylogenetic systematics of the genus Echinococcus (Cestoda: Taeniidae). Int J Parasit. (2013) 43:1017–29. doi: 10.1016/j.ijpara.2013.06.002 23872521

[23] Romig T Ebi D Wassermann M . Taxonomy and molecular epidemiology of Echinococcus granulosus sensu lato. Vet Parasitol. (2015) 213:76–84. doi: 10.1016/j.vetpar.2015.07.035 26264250

[24] Kuchenmüller T Abela-Ridder B Corrigan T Tritscher A . World Health Organization initiative to estimate the global burden of foodborne diseases. Rev Sci Tech Off Int Epizoot. (2013) 32:459–67. doi: 10.20506/rst.32.2.2249 24547649

[25] Brunetti E Kern P Vuitton DA Writing Panel W-I . Expert consensus for the diagnosis and treatment of cystic and alveolar echinococcosis in humans. Acta Trop. (2010) 114:1–16. doi: 10.1016/j.actatropica.2009.11.001 19931502

[26] Schipper HG Kager PA . Diagnosis and treatment of hepatic echinococcosis: an overview. Scand J Gastroenterol. (2004) 39:50–5. doi: 10.1080/00855920410011004 15696850

[27] Armiñanzas C Gutiérrez-Cuadra M Fariñas MC . Hydatidosis: epidemiological, clinical, diagnostic and therapeutic aspects. Rev Esp Quimioter. (2015) 28:116–24. 26032995

[28] Sako Y Nakao M Nakaya K Yamasaki H Ito A . Recombinant antigens for serodiagnosis of cysticercosis and echinococcosis. Parasitol Int. (2006) 55 Suppl:S69–73. doi: 10.1016/j.parint.2005.11.011 16352461

[29] Wassermann M Mackenstedt U Romig T . A loop-mediated isothermal amplification (LAMP) method for the identification of species within the Echinococcus granulosus complex. Vet Parasitol. (2014) 200:97–103. doi: 10.1016/j.vetpar.2013.12.012 24418600

[30] Basturk O Hong SM Wood LD Adsay NV Albores-Saavedra J Biankin AV . A revised classification system and recommendations from the Baltimore consensus meeting for neoplastic precursor lesions in the pancreas. Am J Surg Pathol. (2015) 39:1730–41. doi: 10.1097/pas.0000000000000533 26559377 PMC4646710

[31] Liu J Qu S Zhang T Gao Y Shi H Song K . Applications of single-cell omics in tumor immunology. Front Immunol. (2021) 12:697412. doi: 10.3389/fimmu.2021.697412 34177965 PMC8221107

[32] Labani-Motlagh A Ashja-Mahdavi M Loskog A . The tumor microenvironment: a milieu hindering and obstructing antitumor immune responses. Front Immunol. (2020) 11:940. doi: 10.3389/fimmu.2020.00940 32499786 PMC7243284

[33] Chen TY You L Hardillo JAU Chien MP . Spatial transcriptomic technologies. Cells. (2023) 12(16):2042. doi: 10.3390/cells12162042 37626852 PMC10453065

[34] Pan Y Lu F Fei Q Yu X Xiong P Yu X . Single-cell RNA sequencing reveals compartmental remodeling of tumor-infiltrating immune cells induced by anti-CD47 targeting in pancreatic cancer. J Hematol Oncol. (2019) 12:124. doi: 10.1186/s13045-019-0822-6 31771616 PMC6880569

[35] Sun H Zhang D Huang C Guo Y Yang Z Yao N . Hypoxic microenvironment induced spatial transcriptome changes in pancreatic cancer. Cancer Biol Med. (2021) 18:616–30. doi: 10.20892/j.issn.2095-3941.2021.0158 34086429 PMC8185871

[36] Huang H Brekken RA . Recent advances in understanding cancer-associated fibroblasts in pancreatic cancer. Am J Physiol Cell Physiol. (2020) 319:C233–43. doi: 10.1152/ajpcell.00079.2020 32432930 PMC7500219

[37] Zhang Y Wang D Peng M Tang L Ouyang J Xiong F . Single-cell RNA sequencing in cancer research. J Exp Clin Cancer Res. (2021) 40:81. doi: 10.1186/s13045-023-01494-6 33648534 PMC7919320

[38] Han X Wang R Zhou Y Fei L Sun H Lai S . Mapping the mouse cell atlas by microwell-seq. Cell. (2018) 172:1091–1107.e17. doi: 10.1016/j.cell.2018.02.001 29474909

[39] Mourglia-Ettlin G Marqués JM Chabalgoity JA Dematteis S . Early peritoneal immune response during Echinococcus granulosus establishment displays a biphasic behavior. PloS NeglTrop Dis. (2011) 5:e1293. doi: 10.1371/journal.pntd.0001293 21912714 PMC3166041

[40] Pan W Zhou HJ Shen YJ Wang Y Xu YX Hu Y . Surveillance on the status of immune cells after Echinnococcus granulosus protoscoleces infection in Balb/c mice. PloS One. (2013) 8:e59746. doi: 10.1371/journal.pone.0059746 23555767 PMC3608569

[41] Gottstein B Soboslay P Ortona E Wang J Siracusano A Vuitton D . Immunology of alveolar and cystic echinococcosis (AE and CE). Adv Parasitol. (2017) 96:1–54. doi: 10.1111/j.1399-0039.1998.tb02275.x 28212788

[42] Vuitton DA . The ambiguous role of immunity in echinococcosis: protection of the host or of the parasite? Acta Trop. (2003) 85:119–32. doi: 10.1016/s0001-706x(02)00230-9 12606089

[43] Zhang QM He Y Luo N Patel SJ Han YJ Gao RR . Landscape and dynamics of single immune cells in hepatocellular carcinoma. Cell. (2019) 179:829–+. doi: 10.1016/j.cell.2019.10.003 31675496

[44] Walker JA McKenzie AN . Development and function of group 2 innate lymphoid cells. Curr Opin Immunol. (2013) 25:148–55. doi: 10.1016/j.coi.2013.02.010 23562755 PMC3776222

[45] Thorsson V Gibbs DL Brown SD Wolf D Bortone DS Ou Yang TH . The immune landscape of cancer. Immunity. (2018) 48:812–830.e14. doi: 10.1016/j.immuni.2021.01.011 29628290 PMC5982584

[46] Jiang XF Zhang XF Jiang N Sun YT Li T Zhang J . The single-cell landscape of cystic echinococcosis in different stages provided insights into endothelial and immune cell heterogeneity. Front Immunol. (2022) 13:13. doi: 10.3389/fimmu.2022.1067338 36569953 PMC9772464

[47] Reinehr M Micheloud C Grimm F Kronenberg PA Grimm J Beck A . Pathology of echinococcosis: a morphologic and immunohistochemical study on 138 specimens with focus on the differential diagnosis between cystic and alveolar echinococcosis. Am J Surg Pathol. (2020) 44:43–54. doi: 10.1097/pas.0000000000001374 31567204

[48] Torgerson PR Schweiger A Deplazes P Pohar M Reichen J Ammann RW . Alveolar echinococcosis: from a deadly disease to a well-controlled infection. Relative survival and economic analysis in Switzerland over the last 35 years. J Hepatol. (2008) 49:72–7. doi: 10.1016/j.jhep.2008.03.023 18485517

[49] Jiang T Sun W Aji T Shao Y Guo C Zhang C . Single-cell heterogeneity of the liver-infiltrating lymphocytes in individuals with chronic Echinococcus multilocularis infection. Infect Immun. (2022) 90:e0017722. doi: 10.1128/iai.00177-22 36317875 PMC9670881

[50] Zhang N Bevan MJ . CD8(+) T cells: foot soldiers of the immune system. Immunity. (2011) 35:161–8. doi: 10.1016/j.immuni.2011.07.010 21867926 PMC3303224

[51] Chauchet A Grenouillet F Knapp J Richou C Delabrousse E Dentan C . Increased incidence and characteristics of alveolar echinococcosis in patients with immunosuppression-associated conditions. Clin Infect Dis. (2014) 59:1095–104. doi: 10.1093/cid/ciu520 25034426

[52] Zhang C Lin R Li Z Yang S Bi X Wang H . Immune exhaustion of T cells in alveolar echinococcosis patients and its reversal by blocking checkpoint receptor TIGIT in a murine model. Hepatology. (2020) 71:1297–315. doi: 10.1002/hep.30896 31410870

[53] Wang M Deng B Jiang T Duolikun A Li Y Ainiwaer A . Upregulation of CD244 promotes CD8(+) T cell exhaustion in patients with alveolar echinococcosis and a murine model. Parasit Vectors. (2024) 17:483. doi: 10.1186/s13071-024-06573-2 39578914 PMC11585139

[54] Gharbi HA Hassine W Brauner MW Dupuch K . Ultrasound examination of the hydatic liver. Radiology. (1981) 139:459–63. doi: 10.1148/radiology.139.2.7220891 7220891

[55] Pohnan R Ryska M Hytych V Matej R Hrabal P Pudil J . Echinococcosis mimicking liver Malignancy: a case report. Int J Surg Case Rep. (2017) 36:55–8. doi: 10.1016/j.ijscr.2017.04.032 28531871 PMC5440282

[56] Ali-Khan Z Siboo R Gomersall M Faucher M . Cystolytic events and the possible role of germinal cells in metastasis in chronic alveolar hydatidosis. Ann Trop Med Parasitol. (1983) 77:497–512. doi: 10.1080/00034983.1983.11811742 6660955

[57] Eckert J Thompson RC Mehlhorn H . Proliferation and metastases formation of larval Echinococcus multilocularis. I. Animal model, macroscopical and histological findings. Z Parasitenkd. (1983) 69:737–48. doi: 10.1007/BF00927423 6659651

[58] Buttenschoen K Kern P Reuter S Barth TF . Hepatic infestation of Echinococcus multilocularis with extension to regional lymph nodes. Langenbecks Arch Surg. (2009) 394:699–704. doi: 10.1007/s00423-009-0481-0 19373487

[59] Amano T Hayashi S Nishida T Matsubara T Takahashi K Nakamatsu D . Alveolar echinococcosis mimicking a hepatic neoplasm with lymph node metastasis: a case report. Case Rep Gastroenterol. (2018) 12:587–96. doi: 10.1159/000492461 30386197 PMC6206959

[60] Buttenschoen K Gruener B Carli Buttenschoen D Reuter S Henne-Bruns D Kern P . Palliative operation for the treatment of alveolar echinococcosis. Langenbecks Arch Surg. (2009) 394:199–204. doi: 10.1007/s00423-008-0367-6 18575882

[61] Moriichi K Fujiya M Goto T Okumura T . Echinococcosis infection diagnosed based on the histological findings of a lymph node involvement obtained by EUS-FNA. Endosc Ultrasound. (2018) 7:210–1. doi: 10.4103/eus.eus_90_17 29536956 PMC6032694

[62] Wang Q Cui Y Ren L Wang H Wang Z Wang H . Suspected regional lymph node metastasis in hepatic alveolar echinococcosis: a case report. Iran J Parasitol. (2020) 15:138–41. doi: 10.18502/ijpa.v15i1.2537 PMC7244840 32489386

[63] Polat P Kantarci M Alper F Suma S Koruyucu MB Okur A . Hydatid disease from head to toe. Radiographics. (2003) 23:475–94. doi: 10.1148/rg.232025704 12640161

[64] Carmena D Benito A Eraso E . Antigens for the immunodiagnosis of Echinococcus granulosus infection: an update. Acta Trop. (2006) 98:74–86. doi: 10.1016/j.actatropica.2006.02.002 16527225

[65] Lissandrin R Tamarozzi F Piccoli L Tinelli C De Silvestri A Mariconti M . Factors influencing the serological response in hepatic Echinococcus granulosus infection. Am J Trop Med Hyg. (2016) 94:166–71. doi: 10.4269/ajtmh.15-0219 26503271 PMC4710424

[66] Zhang W McManus DP . Recent advances in the immunology and diagnosis of echinococcosis. FEMS Immunol Med Microbiol. (2006) 47:24–41. doi: 10.1111/j.1574-695x.2006.00060.x 16706785

[67] Cattaneo F Graffeo M Brunetti E . Extrahepatic textiloma long misdiagnosed as calcified echinococcal cyst. Case Rep Gastrointest Med. (2013) 2013:261685. doi: 10.1155/2013/261685 23533840 PMC3600324

[68] Engler A Shi R Beer M Schmidberger J Kratzer W Barth TFE . Simple liver cysts and cystoid lesions in hepatic alveolar echinococcosis: a retrospective cohort study with Hounsfield analysis. Parasite. (2019) 26:54. doi: 10.1051/parasite/2019057 31469072 PMC6716343

[69] Brunetti E Tamarozzi F Macpherson C Filice C Piontek MS Kabaalioglu A . Ultrasound and cystic echinococcosis. Ultrasound Int Open. (2018) 4:E70–8. doi: 10.1002/9781444316841.ch27 30364890 PMC6199172

[70] Brunetti E Junghanss T . Update on cystic hydatid disease. Curr Opin Infect Dis. (2009) 22:497–502. doi: 10.1097/qco.0b013e328330331c 19633552

[71] Zhang W Wen H Li J Lin R McManus DP . Immunology and immunodiagnosis of cystic echinococcosis: an update. Clin Dev Immunol. (2012) 2012:101895. doi: 10.1155/2012/101895 22235225 PMC3253442

[72] Kalantari E Bandehpour M Pazoki R Taghipoor-Lailabadi N Khazan H Mosaffa N . Application of recombinant Echinococcus granulosus antigen B to ELISA kits for diagnosing hydatidosis. Parasitol Res. (2010) 106:847–51. doi: 10.1007/s00436-010-1726-0 20143095

[73] Feng X Wen H Zhang Z Chen X Ma X Zhang J . Dot immunogold filtration assay (DIGFA) with multiple native antigens for rapid serodiagnosis of human cystic and alveolar echinococcosis. Acta Trop. (2010) 113:114–20. doi: 10.1016/j.actatropica.2009.10.003 19836341

[74] Bauomi IR El-Amir AM Fahmy AM Zalat RS Diab TM . Evaluation of purified 27.5 kDa protoscolex antigen-based ELISA for the detection of circulating antigens and antibodies in sheep and human hydatidosis. J Helminthol. (2015) 89:577–83. doi: 10.1017/s0022149x14000479 25006882

[75] Vacirca D Perdicchio M Campisi E Delunardo F Ortona E Margutti P . Favourable prognostic value of antibodies anti-HSP20 in patients with cystic echinococcosis: a differential immunoproteomic approach. Parasite Immunol. (2011) 33:193–8. doi: 10.1111/j.1365-3024.2010.01264.x 21306401

[76] Zhang WB Li J Li Q Yang D Zhu B You H . Identification of a diagnostic antibody-binding region on the immunogenic protein EpC1 from Echinococcus granulosus and its application in population screening for cystic echinococcosis. Clin Exp Immunol. (2007) 149:80–6. doi: 10.1201/b22030-62 PMC1942036 17403055

[77] Marinova I Nikolov G Michova A Kurdova R Petrunov B . Quantitative assessment of serum-specific IgE in the diagnosis of human cystic echinococcosis. Parasite Immunol. (2011) 33:371–6. doi: 10.1111/j.1365-3024.2011.01292.x 21480933

[78] de la Rue ML Yamano K Almeida CE Iesbich MP Fernandes CD Goto A . Serological reactivity of patients with Echinococcus infections (E. granulosus, E. vogeli, and E. multilocularis) against three antigen B subunits. Parasitol Res. (2010) 106:741–5. doi: 10.1007/s00436-009-1707-3 20066435

[79] Hernández-González A Muro A Barrera I Ramos G Orduña A Siles-Lucas M . Usefulness of four different Echinococcus granulosus recombinant antigens for serodiagnosis of unilocular hydatid disease (UHD) and postsurgical follow-up of patients treated for UHD. Clin Vaccine Immunol. (2008) 15:147–53. doi: 10.1128/CVI.00363-07 PMC2223859 17989342

[80] Hajjafari A Sadr S Santucciu C Masala G Bayat M Lotfalizadeh N . Advances in detecting cystic echinococcosis in intermediate hosts and new diagnostic tools: a literature review. Vet Sci. (2024) 11(6):227. doi: 10.3390/vetsci11060227 38921974 PMC11209443

[81] Auer H Picher O Aspock H . Combined application of enzyme-linked immunosorbent-assay (ELISA) and indirect hemagglutination test (IHA) as a useful tool for the diagnosis and postoperative surveillance of human alveolar and cystic echinococcosis. Zentralblatt Fur Bakteriologie Mikrobiologie Und Hygiene Ser a-Medical Microbiol Infect Dis Virol Parasitol. (1988) 270:313–25. doi: 10.1016/s0176-6724(88)80169-x 3223143

[82] Ji J Li B Li J Danzeng W Li J Zhao Y . Comprehensive characterization of plasma cell-free Echinococcus spp. DNA in echinococcosis patients using ultra-high-throughput sequencing. PloS NeglTrop Dis. (2020) 14:e0008148. doi: 10.1371/journal.pntd.0008148 32282820 PMC7209354

[83] Veland N Espinosa D Valencia BM Ramos AP Calderon F Arevalo J . Polymerase chain reaction detection of Leishmania kDNA from the urine of Peruvian patients with cutaneous and mucocutaneous leishmaniasis. Am J Trop Med Hyg. (2011) 84:556–61. doi: 10.4269/ajtmh.2011.10-0556 21460009 PMC3062448

[84] Weerakoon KG McManus DP . Cell-free DNA as a diagnostic tool for human parasitic infections. Trends Parasitol. (2016) 32:378–91. doi: 10.1016/j.pt.2016.01.006 26847654

[85] Baraquin A Hervouet E Richou C Flori P Peixoto P Azizi A . Circulating cell-free DNA in patients with alveolar echinococcosis. Mol Biochem Parasitol. (2018) 222:14–20. doi: 10.1016/j.molbiopara.2018.04.004 29679605

[86] Moradi M Meamar AR Akhlaghi L Roozbehani M Razmjou E . Detection and genetic characterization of Echinococcus granulosus mitochondrial DNA in serum and formalin-fixed paraffin embedded cyst tissue samples of cystic echinococcosis patients. PloS One. (2019) 14:e0224501. doi: 10.1371/journal.pone.0224501 31661532 PMC6818807

[87] Chaya D Parija SC . Performance of polymerase chain reaction for the diagnosis of cystic echinococcosis using serum, urine, and cyst fluid samples. Trop Parasitol. (2014) 4:43–6. doi: 10.4103/2229-5070.129164 24754027 PMC3992803

[88] Wan Z Peng X Ma L Tian Q Wu S Li J . Targeted sequencing of genomic repeat regions detects circulating cell-free Echinococcus DNA. PloS NeglTrop Dis. (2020) 14:e0008147. doi: 10.1371/journal.pntd.0008147 32155159 PMC7083330

[89] Tang J Qin X Hou S Huo Y Wu P Qian B . Alveolar echinococcosis drives functional reprogramming of hepatic CD8(+) T cells. Front Cell Infect Microbiol. (2026) 16:1747682. doi: 10.3389/fcimb.2026.1747682 41798755 PMC12960575

[90] Ou Z Li L Ren P Zhou TT He F Chen J . Spatiotemporal transcriptomic profiling reveals the dynamic immunological landscape of alveolar echinococcosis. Adv Sci (Weinh). (2025) 12:e2405914. doi: 10.1002/advs.202405914 39985260 PMC12079354

[91] Shao Y Xia M Song Y Yan Y Dong X Zong H . Echinococcus multilocularis calreticulin inhibits lectin pathway of complement activation by directly binding to mannose-binding lectin. Pathogens. (2025) 14(4):354. doi: 10.3390/pathogens14040354 40333123 PMC12030537

[92] Zhang C Li T Hou S Tang J Wen R Wang C . Enhancing the therapeutic potential of P29 protein-targeted monoclonal antibodies in the management of alveolar echinococcosis through CDC-mediated mechanisms. PloS Pathog. (2024) 20:e1012479. doi: 10.1371/journal.ppat.1012479 39178325 PMC11376570

[93] Chen L Cheng Z Xian S Zhan B Xu Z Yan Y . Immunization with EmCRT-induced protective immunity against Echinococcus multilocularis infection in BALB/c mice. Trop Med Infect Dis. (2022) 7(10):279. doi: 10.3390/tropicalmed7100279 36288020 PMC9610995

[94] Koziol U Rauschendorfer T Zanon Rodríguez L Krohne G Brehm K . The unique stem cell system of the immortal larva of the human parasite Echinococcus multilocularis. Evodevo. (2014) 5:10. doi: 10.1186/2041-9139-5-10 24602211 PMC4015340

[95] Nian X Li L Ma X Li X Li W Zhang N . Understanding pathogen-host interplay by expression profiles of lncRNA and mRNA in the liver of Echinococcus multilocularis-infected mice. PloS NeglTrop Dis. (2022) 16:e0010435. doi: 10.21203/rs.3.rs-589602/v1 35639780 PMC9187083

[96] Herz M Zarowiecki M Wessels L Pätzel K Herrmann R Braun C . Genome-wide transcriptome analysis of Echinococcus multilocularis larvae and germinative cell cultures reveals genes involved in parasite stem cell function. Front Cell Infect Microbiol. (2024) 14:1335946. doi: 10.3389/fcimb.2024.1335946 38333034 PMC10850878

[97] Lu J Sheng Y Qian W Pan M Zhao X Ge Q . scRNA-seq data analysis method to improve analysis performance. IET Nanobiotechnol. (2023) 17:246–56. doi: 10.1049/nbt2.12115 36727937 PMC10190501

[98] Barth TFE Casulli A . Morphological characteristics of alveolar and cystic echinococcosis lesions in human liver and bone. Pathogens. (2021) 10(10):1326. doi: 10.3390/pathogens10101326 34684275 PMC8537120

[99] Kiselev VY Andrews TS Hemberg M . Challenges in unsupervised clustering of single-cell RNA-seq data. Nat Rev Genet. (2019) 20:273–82. doi: 10.1038/s41576-018-0088-9 30617341

[100] Hicks SC Townes FW Teng M Irizarry RA . Missing data and technical variability in single-cell RNA-sequencing experiments. Biostatistics. (2018) 19:562–78. doi: 10.1093/biostatistics/kxx053 29121214 PMC6215955

[101] Ramsköld D Luo S Wang YC Li R Deng Q Faridani OR . Full-length mRNA-seq from single-cell levels of RNA and individual circulating tumor cells. Nat Biotechnol. (2012) 30:777–82. doi: 10.1038/nbt.2282 Erratum in: Nat Biotechnol. (2020) 38(3):374. doi: 10.1038/s41587-020-0427-1 PMC3467340 22820318

[102] Picelli S Björklund ÅK Faridani OR Sagasser S Winberg G Sandberg R . Smart-seq2 for sensitive full-length transcriptome profiling in single cells. Nat Methods. (2013) 10:1096–8. doi: 10.1038/nmeth.2639 24056875

[103] Hagemann-Jensen M Ziegenhain C Chen P Ramsköld D Hendriks G-J Larsson AJM . Single-cell RNA counting at allele and isoform resolution using smart-seq3. Nat Biotechnol. (2020) 38:708–14. doi: 10.1038/s41587-020-0497-0 32518404

[104] Grün D Kester L van Oudenaarden A . Validation of noise models for single-cell transcriptomics. Nat Methods. (2014) 11:637–40. doi: 10.1038/nmeth.2930 24747814

[105] Tang F Barbacioru C Wang Y Nordman E Lee C Xu N . mRNA-seq whole-transcriptome analysis of a single cell. Nat Methods. (2009) 6:377–82. doi: 10.1038/nmeth.1315 19349980

[106] Hashimshony T Senderovich N Avital G Klochendler A de Leeuw Y Anavy L . CEL-seq2: sensitive highly-multiplexed single-cell RNA-seq. Genome Biol. (2016) 17:77. doi: 10.1186/s13059-016-0938-8 27121950 PMC4848782

[107] Wang X He Y Zhang Q Ren X Zhang Z . Direct comparative analyses of 10X genomics chromium and smart-seq2. Genomics Proteomics Bioinf. (2021) 19:253–66. doi: 10.1016/j.gpb.2020.02.005 33662621 PMC8602399

[108] Macosko EZ Basu A Satija R Nemesh J Shekhar K Goldman M . Highly parallel genome-wide expression profiling of individual cells using nanoliter droplets. Cell. (2015) 161:1202–14. doi: 10.1016/j.cell.2015.05.002 26000488 PMC4481139

[109] Klein AM Mazutis L Akartuna I Tallapragada N Veres A Li V . Droplet barcoding for single-cell transcriptomics applied to embryonic stem cells. Cell. (2015) 161:1187–201. doi: 10.1016/j.cell.2015.04.044 26000487 PMC4441768

[110] Jaitin DA Kenigsberg E Keren-Shaul H Elefant N Paul F Zaretsky I . Massively parallel single-cell RNA-seq for marker-free decomposition of tissues into cell types. Science. (2014) 343:776–9. doi: 10.1126/science.1247651 24531970 PMC4412462

[111] Keren-Shaul H Kenigsberg E Jaitin DA David E Paul F Tanay A . MARS-seq2.0: an experimental and analytical pipeline for indexed sorting combined with single-cell RNA sequencing. Nat Protoc. (2019) 14:1841–62. doi: 10.17504/protocols.io.7hkhj4w 31101904

[112] Zhang P Chen W Tran TN Zhou M Carter KN Kandel I . Thor: a platform for cell-level investigation of spatial transcriptomics and histology. Nat Commun. (2025) 16:7178. doi: 10.1038/s41467-025-62593-1 40764306 PMC12325965

[113] Peruzzu A Mastrandrea S Fancellu A Bonelli P Muehlethaler K Masala G . Comparison and evaluation of analytic and diagnostic performances of four commercial kits for the detection of antibodies against Echinococcus granulosus and multilocularis in human sera. Comp Immunol Microbiol Infect Dis. (2022) 86:8. doi: 10.1016/j.cimid.2022.101816 35472655

[114] Kronenberg PA Reinehr M Eichenberger RM Hasler S Laurimäe T Weber A . Monoclonal antibody-based localization of major diagnostic antigens in metacestode tissue, excretory/secretory products, and extracellular vesicles of Echinococcus species. Front Cell Infect Microbiol. (2023) 13:1162530. doi: 10.3389/fcimb.2023.1162530 37009502 PMC10061086

[115] Kronenberg PA Deibel A Gottstein B Grimm F Müllhaupt B zu Schwabedissen CM . Serological assays for alveolar and cystic echinococcosis-a comparative multi-test study in Switzerland and Kyrgyzstan. Pathogens. (2022) 11:27. doi: 10.3390/pathogens11050518 35631039 PMC9146094

[116] Kellar KL Douglass JP . Multiplexed microsphere-based flow cytometric immunoassays for human cytokines. J Immunol Methods. (2003) 279:277–85. doi: 10.1016/s0022-1759(03)00248-5 12969567

[117] Knapp J Lallemand S Monnien F Felix S Courquet S Umhang G . Real-time multiplex PCR for human echinococcosis and differential diagnosis. Parasite. (2023) 30:3. doi: 10.1051/parasite/2023003 36700708 PMC9886084

